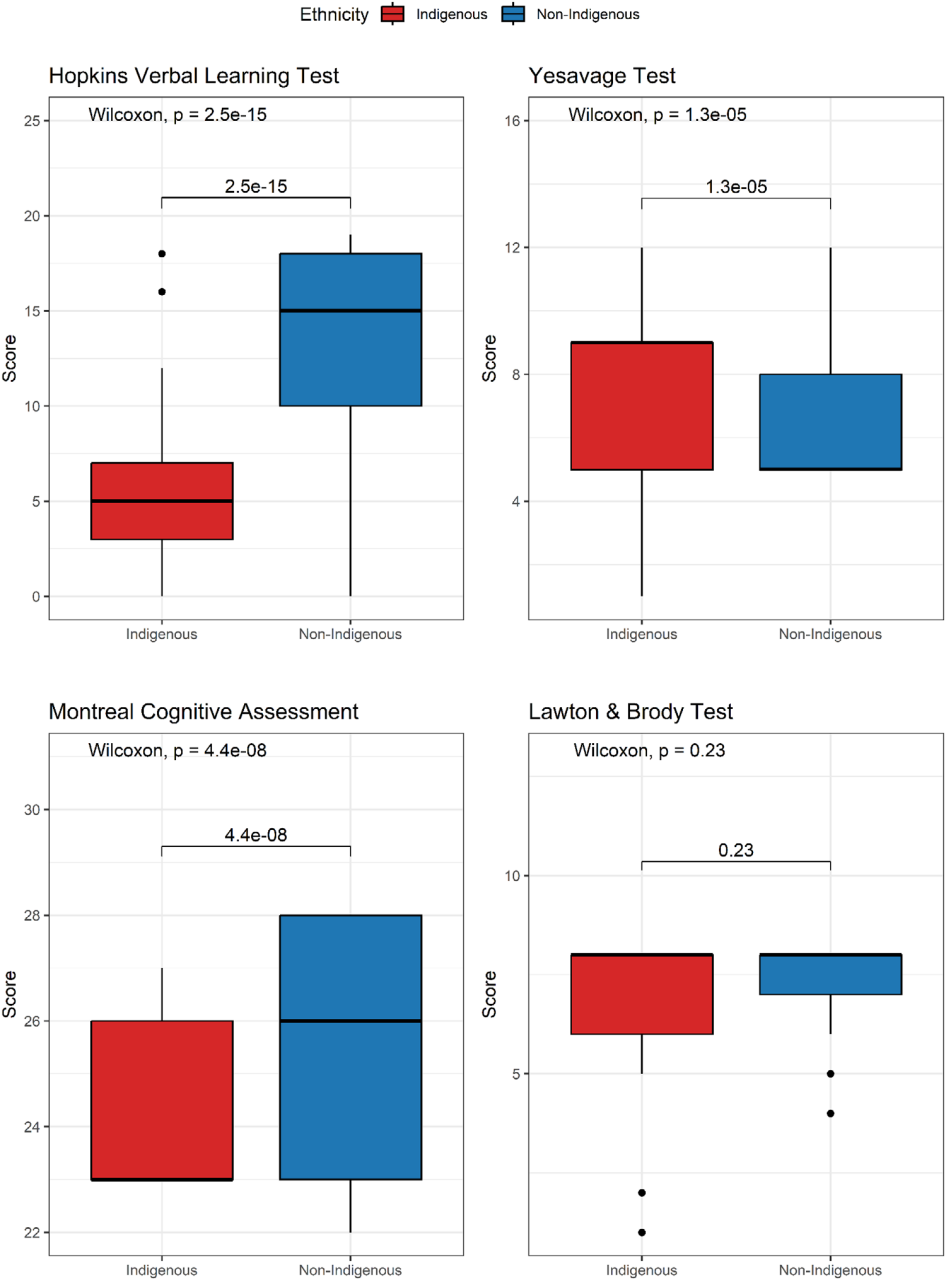# Cognitive Disparities in the Wayuu Indigenous Population: Insights from Neuropsychological Assessment

**DOI:** 10.1002/alz.084777

**Published:** 2025-01-03

**Authors:** Jose Hernandez Preciado, Wanda Torres, Alex Dominguez Vargas, Yesenia Pianneta, Mauricio Medina, Marybel Sinisterra, José Vargas

**Affiliations:** ^1^ Universidad de Maimónides, Buenos Aires Argentina; ^2^ Universidad Simón Bolívar, Barranquilla Colombia; ^3^ Universidad del Norte, Puerto Colombia Colombia; ^4^ Universidad de Caldas, Manizales Colombia

## Abstract

**Background:**

The neuropsychological profile within indigenous communities is a complex interplay of cultural, social, and environmental factors that significantly influence cognitive functioning and distinct neuropsychological patterns. The aim of this study was to assess the neuropsychological profile in the Wayuu indigenous population.

**Methods:**

A cross‐sectional study. Wayuu indigenous individuals (n = 100) and non‐indigenous individuals (n = 100) from the northern coast of the Colombian Caribbean were included. The neuropsychological profile was assessed using the following tests: Hopkins Verbal Learning Test‐Revised (HVLT‐R), Clue Recall, Delayed Recall, Memory Complaint Scale (MCS), Trail Making Test Part A and B (TMT‐A/B), Symbol‐Digit test, Lawton & Brody, Yesavage, and Montreal Cognitive Assessment (MoCA). The Wilcoxon test was applied to compare median scores between Wayuu indigenous and non‐indigenous individuals. Additionally, the Spearman test was utilized to assess the correlation between the scores obtained for each test.

**Results:**

The Wayuu population exhibited significantly lower scores in neuropsychological tests related to memory, learning, autonomy, depression, and cognitive function in comparison to non‐indigenous individuals (p<0.01) (Figure 1). Significant positive correlations were observed between Clue Recall and HLVT‐R (Spearman r = 0.82, p<0.001), Clue Recall and Delayed Recall (Spearman r = 0.69, p<0.001), and between Lawton & Brody and MOCA (Spearman r = 0.42, p<0.001). In contrast, significant negative correlations were found between Yesavage and MOCA (Spearman r = ‐0.45, p < 0.001), and between Yesavage and Lawton/Brody (Spearman r = ‐0.45, p < 0.001).

**Conclusions:**

Our study emphasizes significant cognitive gaps within the Wayuu indigenous community. The neuropsychological landscape of this indigenous population reveals the impact of historical and contemporary factors on cognitive health, emphasizing the necessity of culturally tailored interventions to improve neurocognitive well‐being in these communities.